# Intensive care unit discharge: mind the gap!

**DOI:** 10.1186/s12871-021-01251-7

**Published:** 2021-02-08

**Authors:** Cristian Deana, Giovanni Sermann, Amato De Monte

**Affiliations:** Anesthesia and Intensive Care 1, Department of Anesthesia and Intensive Care Medicine, Academic Hospital “S. Maria della Misericordia”, Piazzale S. M. della Misericordia, 15, 33100 Udine, Italy

**Keywords:** Critically ill patients, Mortality, Intermediate level of care, ICU discharge

## Abstract

Mortality after intensive care discharge is a hot topic in critical care medicine. Many factors probably play a role: patient’s comorbidities and severity of the disease may have great impact on mortality. However it should be taken into account also the level of care that characterizes the ward in which the patient is discharged to. A soft transition from intensive care units to the other hospital wards is desirable to avoid the traumatic step that the fragile post-ICU patient has to face with.

Letter to the Editor.

We read the work by Hamsen and Colleagues about mortality in severely injured patients with great interest [1].

From their analysis of a large dataset, they concluded that 17.7% of all injured patients admitted to ICU and discharged from the unit alive, nevertheless died during their hospital stay.

The authors proposed several possible explanations to interpret their findings: patient characteristics (older and sicker ones were more likely to die), the type of trauma, the level of intensive treatments received during ICU stay, and the level of patient “determination” (or “will”) to live are proposed to be associated with the probability of in-hospital death.

In our opinion, the level of the intensity of care characterising the ward onto which the patients were discharged was not sufficiently considered in their analysis of the factors associated with mortality probability.

ICUs provide the highest level of care intensity in terms of technology, level of organization, monitoring, organ support and human resources. Around the world, the average nurse to patient ratio in the ICU tends towards 1:1, and is rarely lower than 1:2. This permits the continuous monitoring of a patient so that any changes are detected in almost real time, enabling physicians and nurses to do their utmost in their care and rehabilitation of critically ill patients.

General wards are very different and adhere to care models that differ to those of the ICU: continuous monitoring and the provision of personnel could be inadequate to permit the soft transition of patients from the ICU onto the general ward.

In fact, patients who experience ICU care for a long time require intensive rehabilitation in dedicated units to reduce and treat post-intensive care syndrome (PICS) [2].

This is important for returning autonomous and non-disabled people to society.

Capuzzo et al., in a large European study, demonstrated that the sickest patients admitted to the ICU benefit – in terms of reduced mortality – from the intermediate-level care units (IMCU): patients discharged to the IMCU had a 5% lower probability of mortality [3].

Many possible reasons were proposed to account for this result; however, the primary one seems to be that the IMCU is able to guarantee a smooth treatment path without patients being subjected to any sudden changes in the intensity of their care.

Another interesting finding of the study by Hamsen and Colleagues is that, following ICU discharge, nearly 70% of non-survivor patients died within 8 days.

Similar results were found by Valent and Colleagues, who showed that in a large mixed cohort of ICU patients up to 50% of people aged 80 years or older died after being discharged from the ICU [4].

These results certainly implicate the necessity for further investigations into possible predictive factors linked to failed recovery after ICU discharge.

Today’s reality entails scarcity of resources, meaning that energies are sometimes prioritised towards those with higher chances of overcoming their critical illness.

The COVID-19 pandemic has taught us that during times distinguished by resource constraints and a simultaneous high demand for intensive medical care, doctors are called upon to make choices regarding who to invest in and who to allocate more basic treatments.

Critical care research must try to identify predictive criteria relating to the success or failure of the resuscitation treatment being provided to the patient. Big data sets will hopefully help us identify such criteria in this times when evidence based medicine is highly needed [5].

What we must strive for is the possibility to guarantee maximal levels of care to all critically ill patients in ICU, and to avoid transferring them to a lower level of care too early. So, please, mind the gap!

## Data Availability

Not applicable.

## Abbreviations

ICU: Intensive care unit
PICS: Post intensive care syndrome
IMCU: Intermediate care unit
COVID-19: Coronavirus
